# Textual analysis in suicidal crisis management. Clinical case report and proposal of an intervention methodology

**DOI:** 10.3389/fpsyg.2026.1749268

**Published:** 2026-02-05

**Authors:** Jessica Neri, Gian Piero Turchi, Antonio Iudici

**Affiliations:** 1 Institute of Psychology and Psychotherapy, Padua, Italy; 2 Department of Philosophy, Sociology, Education and Applied Psychology, University of Padua, Padua, Italy

**Keywords:** clinical case report, MADIT methodology, suicidal crisis, suicidal emergency, textual analysis

## Abstract

Socioeconomic crises can deeply affect personal narratives and community dynamics, sometimes leading to acute mental health emergencies such as suicidal crises. These situations require structured interventions. Textual analysis, by addressing interactive and narrative dimensions, offers a valuable framework for crisis management and health promotion. This case report describes an individual whose economic and employment instability escalated into an acute suicidal emergency, marked by high and imminent risk, loss of agency, dependence on others, and intensifying suicidal ideation. The intervention employed the MADIT (Methodology of Computerized Analysis of Textual Data), using textual analysis to identify linguistic markers of suicidal intent, guide therapeutic dialog, and support clinical decision-making. The case outlines the user’s texts, the professional’s analysis of underlying narratives, ongoing and final assessments, and the therapeutic measures applied. It also includes the professional’s self-reflection on their engagement with the patient. Examining narrative processes enabled the design, monitoring, and evaluation of an effective intervention strategy. This approach values both the patient’s expressed content and the interactive process through which meaning is constructed in the therapeutic relationship, contributing to more effective crisis management. The case highlights the potential of textual analysis-based methodologies in managing suicidal emergencies, enhancing assessment accuracy, strengthening intervention, and promoting individual health and social cohesion. Overall, this structured yet flexible framework supports anticipation, monitoring, and management of biographical developments within emergency clinical contexts.

## Introduction

In recent years, increasing attention has been devoted to emergencies and their implications for individual and collective functioning. International debate has highlighted how different emergencies require coordinated, network-based interventions involving multiple services and institutional actors (Kohrt and Carruth, 2022; Th’ng et al., 2022; Wang et al., 2022). Examples include territorial and climatic emergencies (Çıplak, 2022; Dolchinkov, 2024; Grubesic and Durbin, 2022; Jiang et al., 2022; Mavroulis et al., 2022; Palmer et al., 2022), emergencies related to war, conflict, or riots (Canavan and Ide, 2024; Hăisan et al., 2022; Spagnolello et al., 2022; Stene et al., 2022), and health-related emergencies (Belita et al., 2022; Cervantes et al., 2023; Parker et al., 2023; Scott et al., 2023). Alongside collective crises, urgency may also involve individuals and their biographical paths (Bornheimer et al., 2023; Callus, 2023; Chrzan-Dętkoś and Walczak-Kozłowska, 2022; Levi-Belz et al., 2022).

Suicidal crises represent a serious public health problem that requires attention and targeted interventions. These are acute crises of intense psychological suffering that destabilize people’s biographies (O'Connor and Nock, 2014; Turecki and Brent, 2016). Biographical emergencies emerge when sudden life changes generate configurations perceived as critical or unmanageable, sometimes culminating in suicidal ideation (Klonsky et al., 2016). It is necessary to develop methodological tools capable of detecting and capturing acute experiences of desperation as they occur, allowing these observations to be translated into intervention practices generalizable beyond the single case (Levi-Belz et al., 2022; Lu et al., 2024).

This clinical case aims to show how textual analysis - using the MADIT, Methodology of Computerized Analysis of Textual Data (Turchi and Orrù, 2014) - can be used to manage suicidal crises in a clinical setting. The clinical scope is to provide practical tools for individuals who may encounter biographical emergencies.

## Methodology of intervention

Within an interactionist paradigm, this theoretical background conceptualizes reality as interactively constructed and therefore mutable and inherently uncertain (Iudici et al., 2019; Neri et al., 2022; Turchi et al., 2022). Emergencies - especially biographical ones - are here understood as interactive configurations involving the individual, their relational network, and the Community. A biographical emergency arises when individuals perceive no viable alternatives for continuing their life narrative, sometimes considering suicide, within personal, relational, social, and institutional systems that may sustain a scenario of death rather than alternative configurations (Turchi et al., 2019; Turchi et al., 2021).

A key distinction is made between urgency, tied to specific demands, and emergence, referring to dialogical processes that make situations critical. Managing emergencies requires acting on interactive processes that shape community cohesion. Psychological intervention therefore shifts from assistance to a Service Architecture that promotes health, interaction, and shared responsibility (Romanelli et al., 2025).

Within this theoretical framework, the methodology used for the present case is part of textual and discourse analysis which operationalizes the principles of interactional and discursive construction of reality through the formalization of ordinary language (Iudici and Corsi, 2017; Iudici et al., 2020; Romanelli et al., 2025).

Drawing on Foucault (1963), Wittgenstein (1953), Harré and Gillett (1994), and Salvini (1994), Methodology of Computerized Analysis of Textual Data (MADIT) (Turchi and Orrù, 2014) views discourse as the process through which configurations of reality are generated through ordinary language. Specifically, texts are analyzed through rhetorical and argumentative structures to detect linguistic patterns shaping experience and agency. These linguistic patterns are formalized into Discursive Repertoires (DRs), defined as sets of rules that describe how meaning is generated in interaction and how they produce a transformative impact on the discursive configurations emerging within the dialogic process (Iudici and Renzi, 2015). DRs are grouped into three classes: *Generative DRs* encompasses narratives that introduce changes within the other’s narrative system. They promote new configurations of meaning and facilitate change. *Stabilisation DRs* consist of narratives that reinforce the narrative organization brought by the individual. They preserve existing realities. *Hybrid DRs* include narratives capable of assuming different orientation depending on the interplay among the various DRs present in narrative (Turchi and Orrù, 2014). The description of each DR is provided in the Appendix, in accordance with its processual properties and its positioning within the three classes.

MADIT defines a Semi-radial Periodic Table of Discursive Repertoires (Turchi et al., 2021), which organizes the 24 DRs according to their properties of meaning generation (Supplementary File) and specific indicators able to measure the configuration (Turchi et al., 2021, 2022; Turchi and Orrù, 2014).

The processual properties that organize DRs highlight not the semantic content of what is said, but the expressive modalities used in language and their impact on interaction. Each DR is associated with the indicator of dialogic weight (dw), a numerical value reflecting its rhetorical–argumentative properties and its capacity to influence discursive interaction, and to open the configuration to other possibilities.

Along the Semi-radial Periodic Table, DRs range from lower to higher dialogic weight: a low dw corresponds to assertive, certain language based on personal and absolute references (e.g., maintenance DRs), whereas a high dw promotes open, generative, and possibilistic interactions grounded in shared elements (e.g., generative DRs) (Turchi et al., 2021).

The Semi-radial Periodic Table of DRs also represents two broader epistemic modalities - affirmation and assertion - showing that greater use of descriptive and shareable references supports assertions (scientific sense) rather than affirmations (common-sense). Finally, the vertical axis reflects the historical evolution of discursive modalities, from earlier forms to more complex ones developed over the phylogenesis of language (Turchi and Orrù, 2014). Referring to the Table, and to the processual properties and dialogic weight that define each repertoire, is useful for assessing the impact of linguistic modalities on the construction of the suicidal crisis configuration and its intervention.

By identifying linguistic markers of closure or openness and their dialogic weight - that is, their contribution to shaping and transforming a given situation or configuration - this approach enables timely intervention before critical points solidify, supporting the management of emergent situations and fostering alternative trajectories oriented toward health and social cohesion.

The process of analysis and intervention follows specific operational steps (Turchi and Orrù, 2014):

1. Demand analysis – identifying the client’s request and underlying needs.
2. Configuration analysis – detecting the dominant discursive repertoires and their implications.
3. Transforming the urgency into emergence – observing the cognitive process (need) underlying the user’s request.
4. Definition and sharing of objectives – establishing goals that are collaboratively defined and operational.
5. Definition of Strategies – outlining clinical interventions and corresponding actions.
6. Evaluation – monitoring changes in the discursive configuration between T0 and T1 using measurable process and outcome indicators.

By integrating this specialized methodology of textual analysis with clinical decision-making through structured operational steps, MADIT supports the transformation of biographical emergencies into emergent processes, fostering the co-construction of meaning configurations oriented toward health, agency, and autonomous management (Iudici et al., 2020; Iudici et al., 2019; Orrù et al., 2024).

## The clinical case

In line with the methodology described above, psychotherapeutic counseling in biographical emergencies aims to activate and develop the roles and skills embedded in the client’s biography. This case concerns a counseling process centered on a discursive configuration in which suicide appeared as the only viable option and an imminent threat. Key issues included job loss and a perceived lack of institutional and social support, constructed as irreversible and unchangeable.

The patient, a man in his early sixties, was facing a severe personal crisis marked by economic and employment instability that had persisted for nearly two years and had escalated into an acute suicidal emergency. He presented a high and imminent suicide risk, with pervasive loss of agency, dependency in decision-making, and intensifying suicidal ideation. The patient’s clinical background includes substance use, recurrent job loss, a recent divorce, and reduced contact with his adult children. Over the past ten years, he had sporadic and time-limited contact with public addiction services, characterized by non-continuous care that was discontinued following the completion of basic assessments and medical visits required for the reinstatement of his driver’s license.

From a family perspective, the patient has two children, with whom contact is limited and largely initiated by the father. Although child support payments are maintained, they are sustained with significant financial difficulty. An informal social network is present and represents a relevant support resource, enabling the patient to meet daily needs through loans and practical assistance from friends.

No additional structured interventions were identified. Overall, the patient’s care trajectory appears fragmented, with a prevailing pattern of autonomous functioning and selective engagement with services, primarily oriented toward addressing specific needs rather than sustained or integrated care pathways.

### Identifying details have been omitted for confidentiality

The case is analyzed through the intervention process and the clinical strategies adopted, focusing on how these interacted with the patient’s discursive configurations over time.

**Figure 1:**
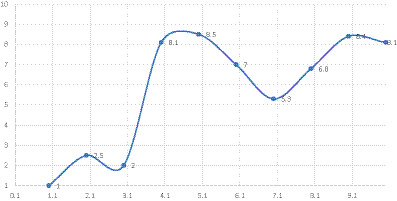
Timeline of the primary episodes and clinical interventions of care. X-axes = number of clinical interventions listed in Table 1. Y-axes = use of discursive repertoires with varying orientations toward health (dw).

With reference to the methodological elements described and resumed in the Semi-radial Periodic Table of Discursive Repertoires, Figure 1 presents a chronological timeline of the episode of care, illustrating the relationship between successive clinical interventions and changes in the dialogic weight of the emergent configuration, as determined by the predominant DRs used at each phase. Prevalent moments of shift are considered in the use of discursive modalities more or less oriented toward health, ranging from a low level of health (dw = 0.1) to a high level of health (dw = 9.9).

In the initial stages of the intervention, DRs oriented toward establishing and maintaining a condition of distress were prevalent (e.g., DRs such as Certify Reality; Contraposition), resulting in a configuration characterized by low generativity. As the intervention progressed, clinical actions increasingly promoted generative and health-oriented DRs, corresponding to a progressive increase in dialogic weight (e.g., DRs such as Description, Possibility, Anticipation). This shift is particularly evident in the later phases of the intervention, which focus on strategy development, service integration, and evaluation, culminating in the closure of care.

The X-axis represents the sequential number of clinical interventions, as detailed in Table 1, corresponding to the chronological progression of the episode of care. The Y-axis represents the dialogic weight of the configuration, reflecting the use of DRs with varying orientations toward health. The figure illustrates how changes in clinical intervention processes are associated with shifts in dialogic weight across different phases of care.

**Table 1:** Clinical interventions and episodes of intervention.

Number of clinical intervention (x)	Intervention process	Episodes of the intervention^1^
1	Demand analysis: from suicidal statement to job search as request	1^ meeting
2	Configuration analysis: *“I cannot take it anymore… I say enough”*	2^-3^ meetings
3	From urgency to Emergency: the promotion of management skills	4^ meeting
4	Definition and sharing the objective: toward the construction of a critical management plan	5^ meeting
5	Definition of strategies	6^ meeting
6	*Strategy 1: Facilitate the development of management strategies and related actions as part of the management plan.*	16^– 20^ meeting
7	*Strategy 2: Facilitate the understanding and use of the available network of services and relationships.*	21^-25^ meetings
8	Strategy 3: Promote the development of a generative service architecture, taking into account the main territorial service involved (social services).	16^-25^ meeting (contacts with social services)
9	Evaluation (monitoring)	26^ -29^ meetings
10	Evaluation (T0-T1)	30^ meeting
11	Closure of the intervention	31^ meeting

Table 1 summarizes the clinical interventions and corresponding episodes of care in chronological order, mapping key phases of the intervention, from initial demand analysis to evaluation and closure.

The consultation lasted approximately six months and comprised about 31 interactions, including face-to-face meetings, phone calls, and SMS exchanges. As illustrated in Figure 1 and detailed in Table 1, the initial demand analysis and definition of aims was completed within the first five days, followed by a consultation phase lasting approximately five months, and a final evaluative and closure phase extending over one month. During the initial phase of the intervention, contacts were more frequent and continuous, occurring several times per day during the first weeks, and subsequently decreasing to approximately one to two contacts per week over the first three months. Interactions were then gradually reduced as the patient manifested a more stable positioning within the care and his biographical pathway, corresponding to the emergence of a configuration that could be monitored and evaluated while being managed more autonomously by the patient.

These clinical interventions and the associated changes in DRs and configuration are described in detail in the following sections.

## The therapeutic intervention

Based on the intervention process and phases outlined above, the following sections describe the key clinical interventions and changes guiding the emergent suicidal crisis toward a configuration oriented to biographical development and criticality management competencies.

### Demand Analysis: from suicidal statement to job search as request

During the first contact and the initial agreed-upon session, the therapist begins to identify and explore the discursive configuration. The client reports that this is the first time he has faced such a situation. A narrative emerges characterized by suicidal statements and a marked difficulty in identifying or applying strategies to manage the crisis. He describes himself as someone who had always worked successfully and found fulfillment in his professional life, which he abruptly left, now finding himself without employment or income.

Tearfully, he certified a specific reality, and justified it: *“I cannot take it anymore. I used to be different. I’ve tried everything. I’m desperate.”* He also expresses urgent concerns about his dependent children, who are experiencing serious financial and daily difficulties, adding: *“I have everything I need to end my life… I see no way out.”*

In this phase, the modalities used are oriented toward shaping and maintaining a specific problematic reality (e.g., certifying reality, justificating the situation), solidifying it and framing suicide as the only possible solution. A biographical and interactional configuration emerges that is oriented toward maintaining the problem, sustained by the use of discursive modalities with low dialogic weight, which anticipate difficulties in envisioning or considering alternative possibilities or management strategies, and reflect a low orientation toward health and criticality management.

The therapist explores the situation through open-ended questions, collecting descriptive elements and using strategies to deepen and disrupt the coherence of the narrative. This enables an expansion of the critical point around which the configuration is organized—namely, the perceived impossibility of solving the problem and the belief that suicide is the only viable option. Gradually, the client formulates a more specific request, shifting slightly from suicidal ideation (while still considering it a possibility) and asking for help in finding an employment.

The most relevant change observed is the shift from certifying a problematic reality and a single, definitive solution toward the formulation of a request for support, albeit initially expressed through delegation to others.

### Configuration analysis: *“I cannot take it anymore… I say enough”*

Following the additional sessions, the therapeutic strategy focused on clarifying and defining the counseling pathway. The emerging configuration showed strong narrative coherence built around the perceived impossibility of managing the situation due to unemployment and insufficient institutional support. The Discursive Repertoires (DRs) were predominantly Stabilization DRs, reinforced by Hybrid ones, maintaining a rigid and closed reality (e.g., certify reality to state the problem; cause of action to determine a causal relation between actions and facts; prediction of a certain future; confirmation of what is personally interpreted). A clear tendency toward delegation emerged: the client attributed unemployment and difficulties to external causes—such as the employer or the pandemic—and considered the restoration of his former job as the only solution. Services were seen as obligated to help, and when expectations were unmet, they were judged as ineffective, reinforcing contraposition in discourse.

These discursive modes, combined with comparisons to a more stable past, legitimized suicidal ideation as a perceived solution to financial, employment, and debt-related difficulties, as reflected in statements like: “*No one does anything. They must help me! … I say enough.”* The client’s view of services was fragmented, with intervention domains conflated and conceived through personal meanings rather than institutional roles. Since discursive configurations are co-constructed, some services also responded through personal interpretations - sometimes judging the client as manipulative or demanding - thus reinforcing the crisis.

From an anticipatory standpoint, the scenario suggested a risk of increasing social marginalization: the client tended to interact with services through delegation and contraposition, while services often relied on verification and control, focusing on urgency rather than supporting competence and shared responsibility over time.

### From urgency to emergency: the promotion of management skills

Considering the discursive configuration and the anticipation of possible critical scenarios emerging from the interaction among different voices, the primary need that can be identified is the promotion of criticality management skills, including within the interface between the client and territorial services.

The process implemented made it possible not to remain at the surface level of the request (to solve a problem/to restore an ideal condition) but to explore it in depth, identifying the cognitive process that generates it as a precipitate. Transforming urgency into emergency therefore requires addressing these interconnected aspects, enabling the counseling pathway to pursue its broader goal: the promotion of Community Health and Social Cohesion. This shift represents a key intervention process and a relevant phase within the therapeutic pathway, enabling a move from a content-focused level (e.g., the explicit request) to work on the discursive and interactional modalities that contribute to maintaining a problematic reality, toward the identification of underlying needs. In this case, this involved promoting management competencies that made it possible to envision additional possibilities and subsequently define management strategies oriented toward continuity and development of the patient’s biographical trajectory and relationships with others.

### Definition and sharing the objective: toward the construction of a critical management plan

In the first interlocutions, the therapeutic work focused on disrupting discursive modes that generated statements about suicide or *not making it*, framed through prescription and delegation. Specific clinical interventions encouraged a comparison between the current situation and the past—described by the client as a time of satisfaction, resilience, and capacity to face adversity while envisioning future goals.

Targeted questions were used to challenge narrative coherence, for example by exploring what had made that past configuration possible and what the same person would do today. This supported anticipation and enabled the formulation of a forward-looking objective: *to build a management plan for the critical areas and aspects prioritized to date.*

The intervention objective thus became more specifically oriented toward constructing a management plan that addressed critical issues, supported the development of related strategies, and promoted the active involvement of the service network. These actions, together with the shared goal, facilitated the emergence of additional Discursive Repertoires (DRs) that progressively made the discursive configuration more flexible and open.

### Strategies of intervention

In planning the intervention, both the clinical and the shared objectives were maintained as reference points to outline strategies that could effectively guide the counseling process.

The selection of intervention strategies was guided by the assessment of the patient’s needs and was implemented once a clearer positioning within the therapeutic (and biographical) pathway emerged, indicating the possibility of transforming the problematic situation into an opportunity for change.

- Strategy 1: Facilitate the development of management strategies and related actions as part of the management plan.
- Strategy 2: Facilitate the understanding and use of the available network of services and relationships.
- Strategy 3: Promote the development of a Generative Service Architecture, taking into account the main territorial service involved (Social Services).

The third strategy represents a step toward building a service network from a generative health perspective, involving dialogue and forms of counseling that include both the client and the services.

However, this paper focuses on the first two strategies, which directly address the biographical emergency. Each strategic line is supported by specific actions and clinical interventions aligned with the defined objective.

#### Strategy 1: facilitate the development of management strategies and related actions as part of the management plan

The first strategy involved using the construction of a management plan as a guiding clinical intervention, structured around the client’s priorities and critical issues. This process made it possible to elicit detailed descriptions of the most pressing concerns, identify priorities requiring intervention, and anticipate possible strategies for addressing them.

The client described the critical issues in concrete terms, such as outstanding payments, limited income, and family-related expenses. Making these elements explicit provided clarity and allowed some disruption of the Stabilization modes sustaining narrative rigidity. As the client expressed: *“Everything is difficult. I am overwhelmed with expenses. I pay one bill, and another notice arrives, then another. The situation is impossible - there is nothing more to be done.”*

By organizing the plan into priority areas with corresponding strategies, the counselor introduced alternative discursive modalities that gradually shifted the configuration from a static conception of impossibility to a more open and actionable perspective, enabling new movements and possibilities for management and change.

In this case, encouraging the patient to evaluate what was most relevant for him—namely, increasing reference to a broader objective (such as being able to do something meaningful for his sons and to “redeem” himself), supported the management of oscillations within the therapeutic process.

#### Strategy 2: facilitate the understanding and use of the available network of services and relationship

The second strategy focused on viewing all active and potential services as co-agents in managing the situation. Given the stabilization modes shaping the client’s relationship with services - especially tendencies toward prescription and delegation - the intervention did not address these patterns directly but concentrated on assessing and clarifying service use as an immediately actionable step. A reflective dialogue helped explore the service network, clarifying the purpose and functions of each service and how to integrate them into the management plan. Once identified, attention shifted to how to interact with them effectively.

At this stage, the client displayed both stabilization repertoires (judgment, contraposition, commentary) and generative repertoires (possibility, anticipation). The intervention prioritized the latter, supporting the perception of service contact as useful in the short and medium term. The practitioner facilitated this orientation by providing contact details, mediating exchanges, or contacting services directly, reinforcing both early self-management and the practitioner’s collaborative stance.

When services failed to meet expectations, discouragement reactivated urgency, expressed in statements such as: *“Services never help me… I’m a failure.”* These episodes were used to challenge static discursive modes and refocus the client on the shared goal, fostering a shift toward active management. Over time, the client’s engagement with services became more autonomous: he anticipated outcomes, evaluated consequences, and moved from immediate problem-solving to a forward-looking management perspective, as shown by statements like, *“I need to let the social worker know I’m making an effort,”* and *“I called her to update her about the payment receipts and my job interview.”*

This highlights how the configuration gradually began to incorporate very specific steps to be enacted personally, re-establishing a degree of responsibility. Narrative modalities such as describing ongoing actions, evaluating what might be more or less useful, and maintaining consistent reference to the shared objective - haracterized by higher dialogic weight in generating alternative realities - supported the transition of the configuration toward a more health-oriented pole.

### Evaluation of change in the discursive configuration

Throughout the counseling process, changes in the discursive configuration and in the client’s actions were continuously observed and anticipated using two process indicators: self-management (Strategy 1) and appropriate service use (Strategy 2). Both indicators showed steady growth, reflecting the implementation of the management plan.

The client gradually became more active in seeking employment, turning to informal networks for work opportunities rather than financial aid, and eventually contacting Social Services independently. Service use became more appropriate: he began asking specific questions, submitting targeted requests, and establishing constructive dialogue with services. He also took responsibility for fulfilling their requests, moving away from critical or oppositional stances. Over time, direct contacts with Social Services increased, along with proposals shared with the therapist and questions about how collaboration could support the management plan.

The client progressively adopted discursive modes oriented toward possibility and action rather than rigidity. Emerging elements included anticipating critical aspects, evaluating possible responses, and identifying which services could address specific issues.

These developments aligned with the outcome indicators defined at the start of the intervention, marking a shift from the initial configuration (T0):

- future-oriented descriptions and anticipations of critical aspects;
- evaluation of how they could be managed;
- reference to the shared goal as a strategic guide.

The adoption of generative repertoires supported two key shifts:

- from delegation to active management;
- toward more appropriate and targeted use of services.

### Closure of the intervention

After a monitoring phase in which several strategies were consolidated, the therapist and client met to review progress, assess the intervention, and consider concluding the counseling. A specific stratagem guided this final meeting: retracing the entire process from the first contact, about five months earlier. The client noted that he had initially felt desperate and without a way out, whereas “now something is moving,” expressing readiness to continue managing his situation.

This reflective comparison highlighted his new positioning and the tools developed. He now evaluated management criteria and strategies, stating that believing change was possible had been crucial. He also emphasized the value of being accompanied, maintaining contact with services, and receiving support from friends and acquaintances. Communication, he noted, enabled collaboration and a more nuanced understanding of service relationships.

Given the shared objective and the progress achieved, counseling ended with a focus on consolidating the skills and strategies developed, seen as resources for present and future challenges. Finally, considering the central role of Social Services, outcomes and tools were shared with them - together with gaps and indicators - to support their continued engagement with the client, who was now actively managing his situation.

Overall, the intervention was associated with a shift toward discursive configurations characterized by higher dialogic weight and a stronger orientation toward health, enabling more generative trajectories of meaning and action.

## Discussion

With this case report is possible to highlight the clinical relevance of a process-oriented, language-based approach to the management of suicidal urgency, while also acknowledging its limitations. A key strength of the case lies in the use of the MADIT methodological framework, which conceptualizes suicidal urgency as an emergent discursive configuration rather than a fixed or purely symptomatic condition. This perspective allows the crisis to be addressed as an opportunity for change and for the resumption of a broader biographical trajectory, beyond immediate stabilization (Turchi et al., 2019).

Through the analysis of Discursive Repertoires and their orientation toward health (with the indicator of dialogical weight), MADIT makes visible how different narrative modalities contribute - as in the present case - to either rigid, delegative constructions of reality or more generative configurations that foster agency, autonomous management, and appropriate and more effective use of services.

Compared with content-focused or risk-factor-based approaches, this framework offers the advantage of capturing the processes through which meanings are constructed and transformed in interaction, thereby guiding clinical interventions in a dynamic and context-sensitive manner (Iudici et al., 2022; Neri et al., 2020). Furthermore, it enables the evaluation of suicidal urgency and intervention not only to prevent suicide risk and ensure physical safety, but also to address the continuity of the biographical and narrative process in which suicidal ideation may emerge (Romanelli et al., 2025; Turchi et al., 2021).

At the same time, the application of this approach involves relevant challenges. In this case, the continuous evaluation of suicidal urgency required close monitoring of dominant discursive repertoires and their evolution over time, as well as careful modulation of interventions to manage oscillations between stabilization-oriented and generative modalities. These difficulties were particularly evident in interactions with territorial services, where delegative and assistentialistic positions tended to intersect.

From a methodological perspective, the reliance on a single case does not allow for statistical generalization of the findings. However, it is possible to underline the chance to model a methodological praxis rather than to generalize outcomes. By illustrating how discursive processes can be systematically analyzed and managed in response to emergent and critical clinical configurations, the case offers transferable insights for similar contexts. Another limitation is related to the application of MADIT which requires specific training to ensure analyses are grounded in a processual conception of language.

Overall, this case underscores both the potential and the demands of a discursive, process-oriented methodology in suicidal crisis management.

## Conclusion

The psychotherapeutic process highlights key elements in managing biographical crises and suicidal emergencies. The case shows how MADIT (Iudici et al., 2022; Turchi and Orrù, 2014) can be applied to urgent situations by interpreting emergencies as discursive and interactional phenomena shaped through language, allowing anticipation and transformation through dialog.

Textual analysis identified the Discursive Repertoires (DRs) sustaining the crisis—mainly Stabilization and Hybrid DRs marked by rigidity, delegation, and loss of agency. Through targeted conversational strategies and ongoing monitoring, the intervention promoted a shift toward Generative DRs, enabling new meanings, anticipations, and management strategies. This shift supported a regained sense of agency, more adequate service use, and better decision-making. MADIT’s linguistic and processual indicators allowed systematic observation and assessment of these changes.

More broadly, the case shows how counseling can promote Community Health and Social Cohesion by fostering interactive and generative practices. Strengthening narrative coherence helped reconfigure the client’s biographical trajectory and mobilize personal and institutional resources toward shared, health-oriented management.

## Data Availability

The original contributions presented in the study are included in the article/Supplementary material, further inquiries can be directed to the corresponding author/s.

## Author’s note

For this psychotherapy process, oral and written informed consent was collected by the client. Sensitive data have been modified to ensure the protection of the client’s privacy and anonymity.
