# Supplementary material for: Textual analysis in suicidal crisis management. Clinical case report and proposal of an intervention methodology
Source: Front Psychol. 2026 Feb 5;17:1749268. doi: 10.3389/fpsyg.2026.1749268 (PMC12917900; doi:10.3389/fpsyg.2026.1749268)

**Appendix A *Glossary Discursive Repertoire (DR) extracted from*** (Moro, Iudici & Turchi, 2025)

| **Discursive Repertoire** | **Description** |
| --- | --- |
| **Stabilisation DRs** | |
| **Certify Reality - CR** | Discursive modality that configures reality by stating a clear, certain and unalterable state of things. The possibility of transformation is unforeseen for this reality. |
| **Opinion - OI** | Discursive modality that configures reality by making explicit that the contents are valid and delimited within the narrator’s own and exclusive perspective. |
| **Cause of Action - CA** | Discursive modality that configures reality through empirical-factual connections of cause-effects with value of truth, which determine an immutable course of events. The argumentation is not epistemologically  founded. |
| **Contraposition - CT** | Discursive modality that configures reality through parallelism between two or more discourse’s parts, which are connected in terms that one excludes the other. The criteria that allow exclusion are not made explicit. |
| **Judgement - JM** | Discursive modality that configures reality according to CR’s processual properties by using moral and/or qualitative attributes without making explicit the criteria used, shaping the narrator’s own and exclusive reality which therefore is not shareable. |
| **Prediction - PV** | Discursive modality that configures realities defining/stating a future scenario as a certain result of the development of a current scenario through a cause-effect rhetorical argumentative link. |
| **Justification - JT** | Discursive modality that configures reality by entailing stabilisation of the “current state of things”: it associates a situation to a previous one in order to legitimize a “state of things”, obstructing the use of other ways to handle or change what is happening. |
| **Non-Answer - NA** | Discursive modality that configures reality in order to avoid the asked question - according to CR’s processual properties - establishing a “state of things” in which the narrator does not adhere properly to the process introduced by the question itself. |
| **Comment - CM** | Discursive modality that configures reality in an inappropriate and irrelevant way to what is asked in the question following the narrator’s  own and exclusive criteria, which are neither made explicit nor sharable. The argumentation does not allow to answer the question asked and it uses CR’s processual properties. |
| **Generalization - GE** | Discursive modality that configures reality by responding inadequately to the question asked and using cross-context argumentations, thus  not covering what is required. The criteria used are not epistemologically founded. |
| **Delegating to others - DE** | Discursive mode that configures reality by delegating to third parties processes that are proper and exclusive to the narrator. |
| **Hybrid DRs** | |
| **Specification - SI** | Discursive modality that configures reality by providing a generation or stabilisation of an explicit and detailed description regarding the configuration it is associated with, limiting its range of application to what is expressed. |
| **Possibility - PS** | Discursive modality that configures reality by using one’s own and exclusive criteria as the only argumentative foundation, without making them explicit and describing them in order to make them shared. It configures reality in probabilistic, possibilistic and uncertain terms. |
| **Confirmation - CP** | Discursive modality that configures reality by validating and supporting what expressed through the Repertory to which it relates. |
| **Implication - IP** | Discursive modality that configures reality shaping the narrator’s own and exclusive position regarding probable situations that could occur and that have not yet occurred, through a cause-effect rhetorical argumentative link. Those situations are reported in a tense (and time) following the one related to the main action (present perfect-simple past or present or future, present future, etc.). |
| **Evaluation - EU** | Discursive modality that configures reality by stating a “state of things” funded on the narrator’s own and exclusive criteria, which, although explicit, are non-sharable. |
| **Declaration of Aims - DA** | Discursive modality that configures reality by transposing the object of the request in a future perspective, without elements of certainty and probability as foundation. |
| **Prescription - PT** | Discursive modality that configures reality as orders/directions given by a third “point of view” position compared to the narrator’s one. Establishes rules and/ or objectives and/or roles to follow, in terms of what one “has to do” or “has not to do”. The argumentation acquires a structure founded on a relation of necessity set by a part of the text. |
| **Reshaping - RS** | Discursive modality that configures realities that limit the generative potential of what the configuration offers. The argumentation’s reference is third and not referable to the narrator. |
| **Generative DRs** | |
| **Description - DS** | Discursive modality that configures reality as a common heritage that does not belong exclusively to any narrator and it needs everyone’s contribution to be maintained. It configures a current or past reality as if the narrator were responding to a question starting with ‘how’ instead of ‘why’ |
| **Targeting - TG** | Discursive modality that configures reality in order to set an objective/purpose/goal to another part of the text, defining actions,  strategies, interventions, etc. Enables the triggering of a discursive configuration aimed at the pursuit of the defined objective/purpose/ goal and, in this way, generating modalities belonging to the generative class and of maximum generative  impact. |
| **Proposal - PP** | Discursive modality that configures uncertain reality, possible in an achievable way and aimed at handling what is requested/offered according to TG’s processual properties. |
| **Consideration - CS** | Discursive modality that configures reality by proposing an argument which uses criteria of analysis that can be shared among several interlocutors, namely that do not belong to any narrators exclusively, but need all of their contribution to maintain them (the criteria). |
| **Anticipation - AT** | Discursive modality that configures reality through an argumentation shaped according to CS’s processual properties. This Repertory configures many different and uncertain situations that can occur and that have not yet occurred using PS’s processual properties. |

Figure 1. Semi-radial Periodic Table of Discursive Repertoires (Turchi et al., 2021; Turchi & Orrù, 2014).


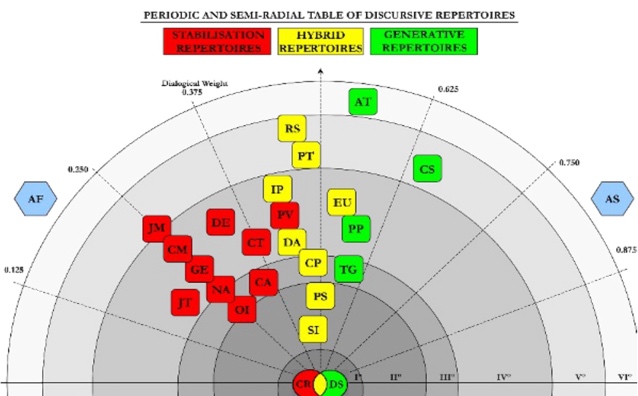

Supplement: Supplementary file 1 [file Supplementary_file_1.docx]
